# Supplementary material for: Reconciling Mining with the Conservation of Cave Biodiversity: A Quantitative Baseline to Help Establish Conservation Priorities
Source: PLoS One. 2016 Dec 20;11(12):e0168348. doi: 10.1371/journal.pone.0168348 (PMC5173368; doi:10.1371/journal.pone.0168348)
Supplement: S1 Dataset — (ZIP) [file pone.0168348.s002.zip › Taxa/Serra Norte/SN_2013/N5S/Lista N5S-66.pdf]

CAVIDADE N5S-0066

Classe	Ordem	Fam/Outros	Gên/Outros	Espécie	S-U	U-S	Zon.
Arachnida	Acari	(Oribatida)		sp.3	X		E/P
Arachnida	Acari	(Parasitengonina)		sp.17		X	E/P
Arachnida	Acari	Asternoseiidae		sp.84	X		E/P
Arachnida	Acari	Ixodidae		jovem	X		E/P
Arachnida	Acari	Laelapidae		sp.3		X	E/P
Arachnida	Amblypygi	Phrynidae	*Heterophrynus*	*longicornis*	6	1	E/P
Arachnida	Araneae	Araneidae		jovem	X	X	E/P
Arachnida	Araneae	Caponiidae		jovem		X	E/P
Arachnida	Araneae	Corinnidae		jovem		2	E/P
Arachnida	Araneae	Ctenidae		jovem		2	E/P
Arachnida	Araneae	Ochyroceratidae		jovem	X	X	E/P
Arachnida	Araneae	Pholcidae	*Mesabolivar*	*aurantiacus*	X	X	E/P
Arachnida	Araneae	Theraphosidae		jovem	1		E/P
Arachnida	Araneae	Theridiidae	*Theridion*	sp.1	X	X	E/P
Arachnida	Araneae	Theridiosomatidae	*Plato*	sp.1	X	X	E/P
Arachnida	Opiliones	Escadabiidae		sp.1		X	E/P
Arachnida	Palpigradi	Eukoeneniidae	*Allokoenenia*	sp.1	X		E/P
Arachnida	Pseudoscorpiones	Bochicidae		sp.1		X	E/P
Arachnida	Pseudoscorpiones	Chernetidae	*Spelaeochernes*	sp.1		X	E/P
Chilopoda	Geophilomorpha	Geophilidae		sp.3	1		E/P
Diplopoda	Polydesmida	Chelodesmidae		jovem	1	1	E/P
Diplopoda	Polydesmida	Pyrgodesmidae		sp.1		X	E/P
Entognatha	Collembola	Entomobryidae		sp.3	X		E/P
Entognatha	Collembola	Paronellidae		sp.1	X		E/P
Entognatha	Diplura	Campodeidae		sp.1	X	X	E/P
Insecta	Blattodea			jovem		1	E/P
Insecta	Coleoptera	Meloidae		sp.1	X		E/P
Insecta	Coleoptera	Scydmaenidae		sp.4	X		E/P
Insecta	Diptera	Chironomidae		sp.	X		E/P
Insecta	Diptera	Psychodidae	Phlebotominae	sp.	X	X	E/P
Insecta	Heteroptera	Reduviidae	Triatominae	sp.		2	E/P
Insecta	Heteroptera	Tingidae		jovem		X	E/P
Insecta	Homoptera	Cixiidae		jovem	X	X	E/P
Insecta	Hymenoptera	Formicidae	*Crematogaster*	sp.1	X		E/P
Insecta	Hymenoptera	Formicidae	*Eurhopalothrix*	sp.1	X	X	E/P
Insecta	Hymenoptera	Formicidae	*Pachycondyla*	sp.2	X	X	E/P
Insecta	Isoptera			sp.	X		E/P
Insecta	Lepidoptera	Tineoidea		sp.1	X	X	E/P
Insecta	Orthoptera	Phalangopsidae	*Paraclodes*	sp.	6		E/P
Insecta	Orthoptera	Phalangopsidae	*Phalangopsis*	sp.	4	22	P
Insecta	Psocoptera	Manicapsocidae	*Nothoentomum*	sp.1	X		E/P
Gastropoda	Pulmonata	Subulinidae		sp.2		X	E/P
Gastropoda	Pulmonata	Subulinidae		sp.5		X	E/P
Malacostraca	Isopoda	Armadillidae		sp.1		X	E/P
Malacostraca	Isopoda	Philosciidae		sp.1		X	E/P
Amphibia	Anura	Strabomantidae	*Pristimantis*	cf. *fenestratus*	2		E/P
Amphibia	Anura			sp.	1	3	E/P
